# ICTV Virus Taxonomy Profile: *Flaviviridae*

**DOI:** 10.1099/jgv.0.000672

**Published:** 2017-01-17

**Authors:** Peter Simmonds, Paul Becher, Jens Bukh, Ernest A Gould, Gregor Meyers, Tom Monath, Scott Muerhoff, Alexander Pletnev, Rebecca Rico-Hesse, Donald B Smith, Jack T Stapleton, ICTV Report Consortium

**Affiliations:** ^1^​ Nuffield Department of Medicine, University of Oxford, Oxford OX1 3SY, UK; ^2^​ Institute of Virology, University of Veterinary Medicine, Hannover D-30559, Germany; ^3^​ Copenhagen Hepatitis C Program (CO-HEP), Copenhagen University Hospital, Hvidovre, Denmark; ^4^​ Unité des Virus Emergents, Faculté de Médecine Timone, 13385 Marseille Cedex 05, France; ^5^​ Institut für Immunologie, Friedrich-Loeffler-Institut, Südufer 10, Greifswald-Riems D-17493, Germany; ^6^​ BioProtection Systems/NewLink Genetics Corporation, 94 Jackson Road, Suite 108, Devens, MA 01434, USA; ^7^​ Abbott Laboratories, 100 Abbott Park Road, Abbott Park, IL 60064-6015, USA; ^8^​ Laboratory of Infectious Diseases, NIAID/NIH, Bethesda, MD 20892, USA; ^9^​ Baylor College of Medicine, Houston, TX 77030-3411, USA; ^10^​ Centre for Immunity, Infection and Evolution, University of Edinburgh, Edinburgh EH9 3FL, UK; ^11^​ Department of Internal Medicine, University of Iowa, Iowa City, IA 52242, USA; ^12^​ Department of Microbiology, University of Iowa, Iowa City, IA 52242, USA

**Keywords:** *Flaviviridae*, taxonomy, ICTV Report

## Abstract

The *Flaviviridae* is a family of small enveloped viruses with RNA genomes of 9000–13 000 bases. Most infect mammals and birds. Many flaviviruses are host-specific and pathogenic, such as hepatitis C virus in the genus *Hepacivirus*. The majority of known members in the genus *Flavivirus* are arthropod borne, and many are important human and veterinary pathogens (e.g. yellow fever virus, dengue virus). This is a summary of the current International Committee on Taxonomy of Viruses (ICTV) report on the taxonomy of the *Flaviviridae*, which is available at www.ictv.global/report/flaviviridae.

## Virion

**Fig. 1.**
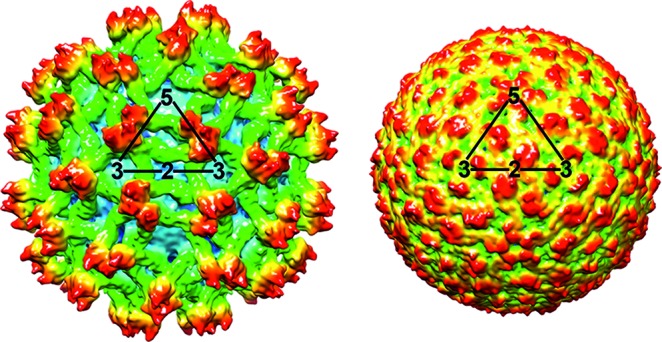
Three-dimensional cryo-electron reconstruction of immature (left) and mature (right) particles of an isolate of dengue virus (courtesy of Richard Kuhn and Michael Rossmann). Shown is a surface rendering of immature dengue virus at 12.5 Å resolution (left) and mature dengue virus at 10 Å resolution (right). The viruses are depicted to scale, but not coloured to scale. Triangles outline one icosahedral unit, with the 2-, 3- and 5-fold axes of symmetry.

Virions are typically spherical in shape with a lipid envelope (Table 1, Fig. 1). Virions have a single, small, basic capsid (C) protein and two (genera *Flavivirus, Hepacivirus* and *Pegivirus*) or three (genus *Pestivirus*) envelope proteins.

**Table 1.** Characteristics of the family *Flaviviridae*

Typical member:	yellow fever virus-D17 (X03700), species *Yellow fever virus*, genus *Flavivirus*
Virion	Enveloped, 40–60 nm virions with a single core protein (except for genus *Pegivirus*) and 2 or 3 envelope glycoproteins
Genome	Approximately 9.0–13 kb of positive-sense, non-segmented RNA
Replication	Cytoplasmic, in membrane vesicles derived from the endoplasmic reticulum (ER); assembled virions bud into the lumen of the ER and are secreted through the vesicle transport pathway
Translation	Directly from genomic RNA containing a type I cap (genus *Flavivirus*) or an internal ribosome entry site (other genera)
Host range	Mammals (all genera); most members of genus *Flavivirus* are arthropod borne
Taxonomy	Currently four genera containing more than 60 species

## Genome

**Fig. 2.**
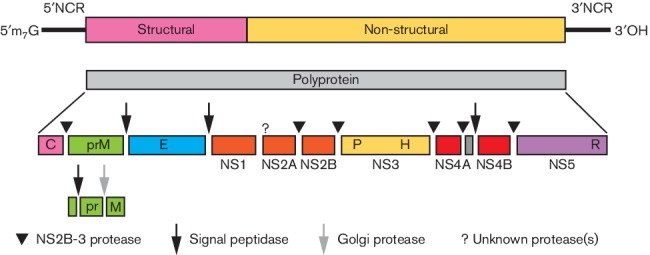
Genome organization and polyprotein processing of members of the genus *Flavivirus*. Boxes below the genome indicate viral proteins generated by proteolytic processing. NCR, non-coding region.

Virus genomes are positive-stranded, non-segmented RNA of approximately 9.2–11, 12.3–13, 8.9–10.5 and 8.9–11.3 kb for members of the genera *Flavivirus*, *Pestivirus*, *Hepacivirus* and *Pegivirus*, respectively (Fig. 2). They contain a single, long ORF flanked by 5′- and 3′-terminal non-coding regions, which form specific secondary structures required for genome replication and translation. Translational initiation of genomic RNA is cap dependent in the case of members of the genus *Flavivirus*, whereas internal ribosome entry site elements are present in members of the other genera.

## Replication

Viral proteins are synthesized as part of a polyprotein that is co- and post-translationally cleaved by viral and cellular proteases. The structural proteins are contained in the N-proximal portion of this polyprotein and the non-structural proteins in the remainder. Replication of members of the family *Flaviviridae* occurs through the synthesis of an antigenome as the template for genome RNA production. Genome RNA also acts as a translational template for the synthesis of viral proteins. Replication complexes are sequestered with a complex topology in membranous structures within the endoplasmic reticulum. Replication enzymes include a serine protease, an RNA helicase and an RNA-dependent RNA polymerase. These proteins are homologous among all members of genus *Flavivirus*, contain conserved motifs and are encoded at similar locations in the genome. Virion assembly, including acquisition of a glycoprotein-containing lipid envelope, occurs by budding through intracellular membranes. Particles are transported in cytoplasmic vesicles through the secretory pathway and released by exocytosis.

## Taxonomy

### Flavivirus

This genus consists primarily of >50 species of arthropod-borne viruses, with distinct groups infecting mosquitoes or ticks [1]. Mammals and birds are the usual primary hosts, in which infections range from asymptomatic to severe or fatal haemorrhagic fever or neurological disease. Important human pathogens include yellow fever virus, dengue virus, Japanese encephalitis virus, West Nile virus and tick-borne encephalitis virus. Other members cause economically important diseases in domestic or wild animals. Additional viruses infecting only arthropods or only mammals (e.g. Tamana bat virus) have been described recently.

### Pestivirus

These viruses infect pigs and ruminants, including cattle, sheep, goats and wild ruminants [2], and are transmitted through contact with infected secretions (respiratory droplets, urine or faeces). Infections may be subclinical or cause enteric, haemorrhagic or wasting diseases, including those by the economically important bovine viral diarrhoea virus and classical swine fever virus.

### Hepacivirus

This genus includes hepatitis C virus, a major human pathogen causing progressive liver disease [3], and several other viruses of unknown pathogenicity that infect horses, rodents, bats, cows and primates [4]. Infections are typically persistent and target the liver.

### Pegivirus

Members are widely distributed in a range of mammalian species, in which they cause persistent infections [5]. To date, they have not been clearly associated with disease.

## Resources

Full ICTV Online (10th) Report: www.ictv.global/report/flaviviridae.

Hepatitis C virus classification: http://talk.ictvonline.org/links/hcv/hcv-classification.html.
